# Screening tool to identify adolescents living with HIV in a community setting in Zimbabwe: A validation study

**DOI:** 10.1371/journal.pone.0204891

**Published:** 2018-10-02

**Authors:** Tsitsi Bandason, Ethel Dauya, Subathira Dakshina, Grace McHugh, Prosper Chonzi, Shungu Munyati, Helen A. Weiss, Victoria Simms, Katharina Kranzer, Rashida Abbas Ferrand

**Affiliations:** 1 Biomedical Research and Training Institute, Harare, Zimbabwe; 2 Harare City Health, Harare, Zimbabwe; 3 MRC Tropical Epidemiology Group, LSHTM, London, United Kingdom; 4 London School of Hygiene and Tropical Medicine, London, United Kingdom; 5 Research Centre Borstel, Borstel, Germany; University of Texas Medical Branch at Galveston, UNITED STATES

## Abstract

**Introduction:**

A simple cost-effective strategy to pre-screen for targeted HIV testing can have substantial benefit in high burden and resource limited settings. A 4-item (previous hospitalisation, orphanhood, poor health status, and recurring skin problems) screening tool to identify adolescents living with HIV has previously shown high sensitivity in healthcare facility settings. We validated this screening tool in a community setting, in Harare, Zimbabwe in a community-based HIV prevalence survey.

**Methods:**

A community-based HIV prevalence survey was conducted among individuals aged 8–17 years with guardian consent and child assent and residing in 7 communities during the period February 2015 to December 2015. Participants without previously diagnosed HIV were evaluated for the probability of having HIV using the screening tool. HIV status was defined using an anonymous HIV test which was done using Oral Mucosal Transudate (OMT). A questionnaire was also administered to ascertain self-reported HIV status and screening tool items. The validity of a 4-item screening tool was tested. Sensitivity and specificity of the screening tool was assessed against the HIV status based on OMT result.

**Results:**

Prevalence survey participants were 5386 children who had an HIV test result, aged 8–17 years. However, 5384, who did not report testing HIV positive and responded to all screening tool item questions were included in the validation. Their median age was 12 (IQR: 10–15) years, 2515 (46.7%) were male. HIV prevalence was 1.3% (95% CI:1.0–1.8%). The 4-item screening tool had poor accuracy with an area under the receiver operating curve of 0.65(95% CI: 0.60–0.72) at a cut-off score≥1. Its sensitivity was 56.3% (95% CI:44.0–68.1%) and specificity of 75.1% (95% CI:73.9–76.3%), PPV of 2.9% (95% CI:2.1–3.9%) and a NPV of 99.2% (95% CI:98.9–99.5%). The number needed to test to diagnose one child using the screening tool was 55% lower than universal testing for HIV.

**Conclusion:**

Use of the 4-item screening tool could be a strategy that can be adopted to identify children living with HIV in a community setting in resource limited settings by reducing the number needed to test compared to universal testing since it is inexpensive, easy to administer and not harmful. However, screening items adapted to a community setting need to be explored to improve the performance of the screening tool.

## Introduction

The coverage of antiretroviral therapy (ART) is lower in children compared to adults (45% vs 54% in 2016) globally, the key reason being high rates of undiagnosed HIV infection in this age group.[1] The World Health Organization (WHO) recommends provider-initiated HIV testing and counselling (PITC) for all individuals attending health-care facilities in high HIV prevalence settings (adult HIV prevalence ≥1%).[2] However, several studies have demonstrated that HIV testing rates among adolescents attending health facilities remain low due to implementation challenges which include unavailability of manpower and testing kits.[3, 4] Community-based strategies are being more widely employed to increase coverage of HIV testing, but the yield is lower than that for facility-based HIV testing.[5] However, community-based universal testing may not be cost-effective in resource-limited settings, particularly among children who have lower HIV prevalence than adults.[6] Using a pre-testing screening tool to identify children at risk of being HIV-positive may increase yield of HIV testing, and be a more efficient and cost-effective approach in resource-limited settings.

We previously developed an HIV screening tool for children and adolescents (aged 6–16 years) at risk of being HIV-positive, who could then be targeted for HIV testing and counselling. The tool, consisting of 4 items (previous hospitalisation, orphanhood, poor health status, and recurring skin problems), with a cut-off score of ≥1 and designed for use in primary care settings by low-cadre, non-professional health workers, had 80% sensitivity and 66% specificity when validated in seven primary health care settings in Harare, Zimbabwe.[7]

In this study, embedded in a community-based HIV prevalence survey, we validated the performance the 4-item screening tool to identify children and adolescents at risk of being HIV-positive in a community setting in Harare, Zimbabwe.

## Materials and methods

### Study population and sampling

During the period February 2015 to December 2015, a cross-sectional HIV prevalence survey was conducted among children aged 8–17 years from all households located in 130 randomly selected Census Enumeration Areas (Zimbabwe Census 2012) in seven urban high density suburbs in south western Harare, Zimbabwe.[8]

### Ethical approval and consent

The study was approved by the Medical Research Council of Zimbabwe and Ethical Review Committees of the London School of Hygiene and Tropical Medicine and the Biomedical Research and Training Institute. Written consent was obtained from all guardians of the participants and participants aged 13 to 17 years and assent from children aged 8 to12 years to respond to all the screening questions and to an anonymous HIV test and referral for a diagnostic HIV.

### Data collection

Participants were asked to respond to an interviewer-administered questionnaire that collected socio-demographic data including the screening tool item questions;

- orphanhood status (Is your mother alive?, Is your father alive?: Yes/No)
- previous hospitalisation (Have you ever been admitted to hospital?: Yes/No)
- recurring skin problems (Have you been having recurring skin problems?: Yes/No)
- poor health (How do you rate your general health?: Excellent, Good, Fair Poor)

Data were collected by trained research assistants on electronic tablets (Nexus 7 2013) using Open Data Kit (ODK) and data transferred to an MS Access database.

Confidential HIV testing was performed by the same research assistants who were trained to collect oral mucosal transudate (OMT) and test using Oraquick HIV-1/2 test kits. The HIV results were recorded on paper forms and the data entered into an MS Access database using Cardiff TELEFORM Intelligent Character Optical Mark Recognition Software (Version 10.9).

### Statistical analysis

All analyses were performed using STATA version 13 (StatCorp, Texas, USA).

The performance of the screening tool was validated after excluding and also after including those who self-reported previously having tested HIV-positive in this analysis. The responses to the screening tool items were coded or recoded as binary (Yes = 1, No = 0, fair/poor health = 1and excellent/good health = 0). The total score was calculated as the sum of the numerical values of the responses to the screening items with a possible minimum score of 0 and maximum score of 4. HIV status was defined based on OMT result and self-reported HIV with documented clinical evidence.

HIV prevalence and the 95% confidence interval estimate was calculated allowing for clustering by community (surburb). The associations between HIV (using the OMT test result) and age, sex, orphanhood status, previous hospitalisation, recurring skin problems and poor health were examined using student t-tests and Chi-squared tests for binomial proportions. Chi-square test for trend was used to check the trend in the prevalence of HIV status by score.

Logistic regression with random effects was used to estimate the odds ratios (OR) and 95% CI associated with HIV status, for each item. Items significantly associated with being HIV-positive (p<0.1) were included in the final multivariable logistic model to be considered in the screening tool for a community setting. Multicollinearity between the screening items in the final model were checked using the logistic regression post estimations programs for data analysis.

Optimum cut-off for the screening tool was checked using a receiver operating characteristic curve (ROC). The area under the curve (AUC) and sensitivity, specificity, positive predictive (PPV) and negative predictive values (NPV) for the screening tool were determined using the OMT HIV test result which was assumed to be the gold standard without error for the purposes of this analysis and also including self-reported HIV status.

The number needed to test to identify one child living with HIV after the application of the screening tool (NNT+) items and score levels was calculated. The reduction in NNT+ compared to universal testing to identify one child living with HIV were determined for different screening items and score levels.

## Results

**Fig 1:**
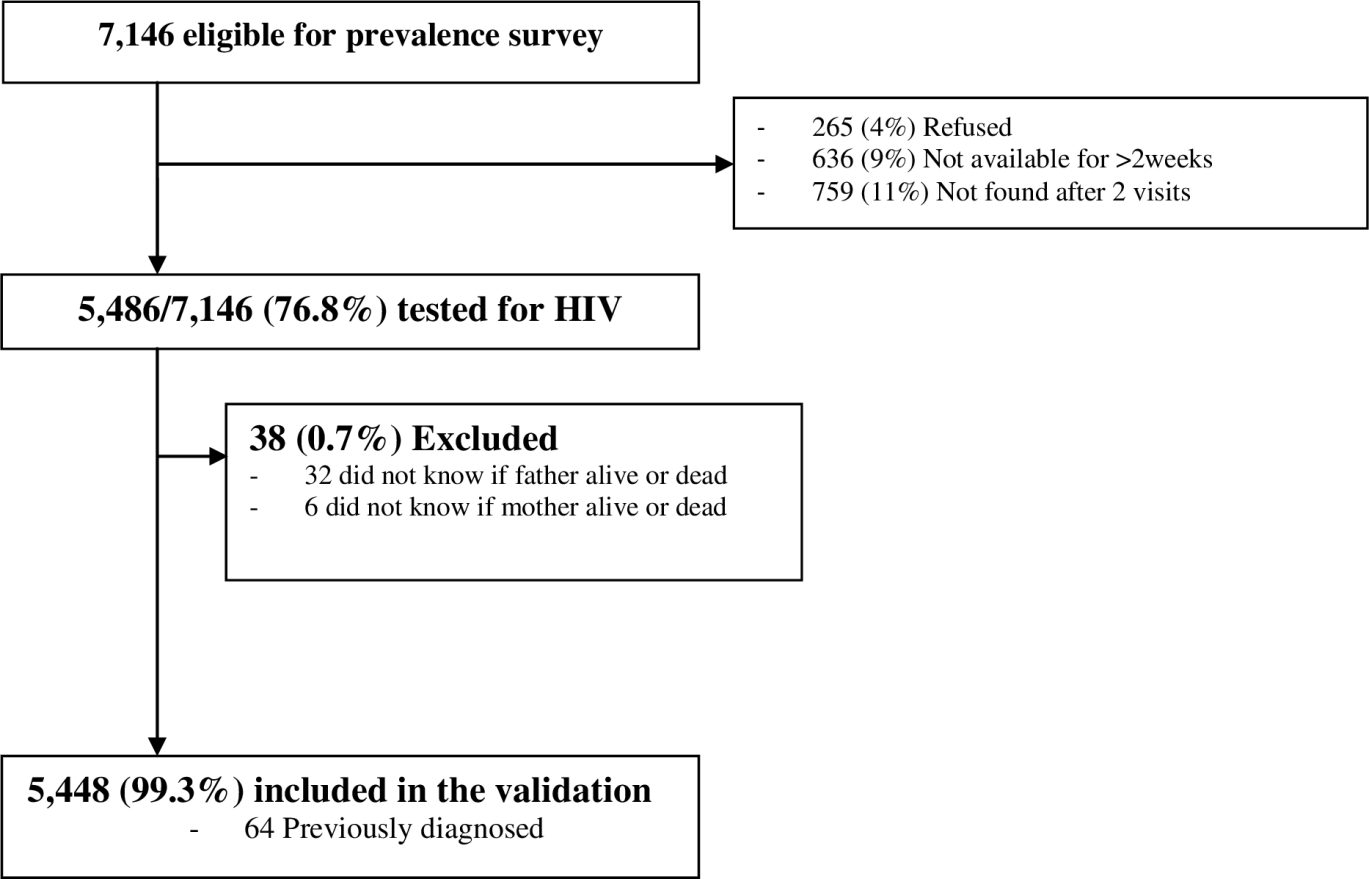
Selection of the validation study sample.

A total of 5486 children aged 8–17 years from 3397 randomly selected households participated in the prevalence survey, with 38 excluded because no information was available on orphanhood status (Fig 1). A total of 64 who self-reported that they were living with HIV were initially excluded from the analysis and of the remaining 5384 participants, 2515 (46.7%) were male, with a median age of 12 years (IQR 10–15) (Table 1). The cluster weighted prevalence of HIV was estimated at 1.3% (95% CI: 1.0–1.8%).

**Table 1:** Baseline characteristics stratified by HIV status.

Characteristic	All	HIV Status		Univariate OR (95% CI)	p-value	Multivariable OR (95% CI)	p-value
		**Positive**	**Negative**				
	**N = 5384**	**n = 71 (%**_**col**_**)**	**n = 5313(%**_**col**_**)**				
**Age (years)**							
8–12 years	2784(51.2%)	27(38.0%)	2757 (51.9%)	1		1	
13–17 years	2600(48.3%)	44 (61.9%)	2556 (48.1%)	1.7(1.2–2.5)	0.002	1.4(0.9–2.2)	0.065
Median age (IQR)	12(10–15)	14(11–16)	12 (10–15)				
**Sex**							
Female	2869(53.3%)	40(56.3%)	2829 (53.2%)	1			
Male	2515(46.7%)	31(43.7%)	2484 (46.7%)	0.9(0.7–1.1)	0.206	Excluded	
**Screening Items**							
Been admitted to hospital before (Yes)	306(5.7%)	11(15.5%)	295 (5.5%)	3.1 (1.9–5.2)	<0.001	2.8(1.6–5.1)	0.001
Had recurring skin problems (Yes)	205(3.8%)	5 (7.0%)	200 (3.8%)	1.9 (1.0–3.8)	0.050	1.4(0.7–2.8)	0.362
One or both parents deceased(Yes)	882(16.4%)	26 (36.6%)	856 (16.1%)	3.0 (2.4–3.8)	<0.001	2.7 (1.9–3.6)	<0.001
Have poor health (Yes)	194(3.6%)	5 (7.0%)	189 (3.5%)	2.0 (1.2–3.5)	0.010	1.4(0.8–2.4)	0.362

P-value: For OR (Odds Ratio)

Using a cut-off of p<0.1, all the screening items and age were significantly associated with testing HIV positive and were included in the multivariable logistic model. However, health status rating and presence of skin problems and age were not associated with testing HIV positive in the multivariable model.

At a cut-off score of ≥1 (confirmed by ROC analysis), the 4-item tool yielded a sensitivity of 56.3% (95% CI:44.0–68.1%) and specificity of 75.1% (95% CI:73.9–76.3%), PPV 2.9% (95% CI: 2.1–3.9%), NPV 99.2% (95% CI: 98.9–99.5%) and area under the curve (AUC) 0.65(95% CI 0.60–0.72).

There was no participant who tested HIV positive who scored 4. The prevalence of HIV increased with screening score, from 0.7%, 3.0%, 1.8% and 9.1% among those scoring 0, 1, 2 and 3 respectively (p<0.001 using chi-square test for trend). A score ≥1 was more likely among children aged 13–17 years compared to those aged 8–12 years (31.9% vs 19.2%, p<0.001) but there was no significant difference by gender (25.4% vs 25.2%, p = 0.817).

The number needed to test to diagnose one child living with HIV using the screening tool was 34 as compared to 76 using universal testing for HIV, which was a reduction of 55% (Table 2). One child would be falsely classified as “not at risk” for every 176 children screened using the tool. Demographic characteristics of those falsely classified showed that 61.3% were female and 54.8% were aged between 13–17 years.

**Table 2:** Properties of 4-item community screening tool excluding participants with previously diagnosed HIV (HIV Prevalence = 1.3%).

		Sn (%) (95% CI)	Sp (%) (95% CI)	PPV (%) (95% CI)	NPV(%) (95% CI)	AUC	NNT+ tool (95% CI)	Reduction in NNT+ compared to universal testing (%)
**Screening Item**								
Been admitted to hospital before	15.5(8.0–26.0)		94.4(93.8–95.1)	3.6(1.8–6.3)	98.8(98.5–99.1)	0.55(0.50–0.59)	28(16–55)	63
Had recurring skin problems	7.0(2.3–15.7)		96.2(95.7–96.7)	2.4(0.8–5.6)	98.7(98.4–99.0)	0.51(0.48–0.55)	41(18–126)	46
One or both parents deceased	36.6(25.5–48.9)		83.9(82.9–84.9)	2.9(1.9–4.3)	99.0(98.7–99.3)	0.60(0.55–0.66)	34(23–52)	55
Have poor health	7.0(2.3–15.7)		96.4(95.9–96.9)	2.6(0.84–5.9)	98.7(98.4–99.0)	0.52(0.49–0.55)	39(17–119)	49
**Screening Tool Score Cut-off**								
≥1	56.3(44.0–68.1)		75.1(73.9–76.3)	2.9(2.1–3.9)	99.2(98.9–99.5)	0.65(0.60–0.72)	34(25–48)	55
≥2	7.0(2.3–15.7)		96.4(95.9–96.9)	2.6(0.8–5.9)	98.7(98.4–99.0)	0.52(0.49–0.55)	39(17–120)	48
≥3	2.8(0.3–9.8)		99.6(99.4–99.8)	8.0(1.0–26.0)	98.7(98.4.99.0)	0.51(0.49–0.53)	13(4–102)	84

Sn -Sensitivity, Sp-Specificity, PPV—Positive Predictive Value, NPV—Negative Predictive Value, NNT+ tool—Number Needed to Test to identify 1 HIV-infected after application of screening tool, AUC:Area Under Curve

To test the performance of the tool using HIV status which includes participants who self-reported and showed documented clinical evidence of living with HIV, the weighted prevalence of HIV was estimated at 2.4% (95% CI: 2.0–2.9%). The screening tool had a sensitivity of 71.4% (95% CI:63.2–78.7%) and specificity of 75.1% (95% CI:73.9–76.3%), PPV 7.0% (95% CI: 5.8–8.5%), NPV 99.0% (95% CI: 98.6–99.3%) and area under the curve (AUC) 0.73 (95% CI 0.70–0.77) (Table 3).

**Table 3:** Properties of 4-item community screening tool including participants with previously diagnosed HIV (HIV Prevalence = 2.4%).

		Sn (%) (95% CI)	Sp (%) (95% CI)	PPV (%) (95% CI)	NPV (%) (95% CI)	AUC	NNT+ tool (95% CI)	Reduction in NNT+ compared to universal testing (%)
**Screening Item**								
Been admitted to hospital before	26.4(19.3–34.5)		94.5(93.8–95.1)	11.2(8.0–15.1)	98.0(97.6–98.4)	0.60(0.57–0.64)	9(7–13)	77
Had recurring skin problems	16.4(10.7–23.6)		96.2(95.7–96.7)	10.3(6.7–15.1)	97.8(97.3–98.1)	0.56(0.53–0.59)	10(7–15)	75
One or both parents deceased	45.7(37.3–54.3)		83.9(82.9–84.9)	6.9(5.4–8.8)	98.3(97.9–98.7)	0.65(0.61–0.69)	14(11–19)	63
Have poor health	20.0(13.7–27.6)		96.4(95.9–96.9)	12.9(8.8–18.1)	97.9(97.4–98.2)	0.58(0.55–0.62)	8(6–11)	80
**Screening Tool Score Cut-off**								
≥1	71.4(63.2–78.7)		75.1(73.9–76.3)	7.0(5.8–8.5)	99.0(98.6–99.3)	0.73(0.69–0.77)	14(12–17)	63
≥2	25.7(18.7–33.8)		96.4(95.9–96.9)	15.9(11.4–21.3)	98.0(97.6–98.4)	0.61(0.57–0.65)	6(5–9)	84
≥3	11.4(6.7–17.9)		99.6(99.4–99.7)	41.0(25.6–57.9)	97.7(95.9–98.1)	0.56(0.53–0.58)	2(2–4)	94

Sn -Sensitivity, Sp-Specificity, PPV—Positive Predictive Value, NPV—Negative Predictive Value, NNT+ tool—Number Needed to Test to identify 1 HIV-infected after application of screening tool AUC-Area Under the Curve

## Discussion

Provider-initiated HIV testing and counselling (PITC) has been recommended by the WHO since 2007 for everyone attending health facilities in high HIV prevalence settings.[9] Facility-based testing relies on an individual attending a health care facility with an illness, and HIV diagnosis therefore often occurs after development of advanced disease.[10, 11] Therefore, community-based strategies are being increasingly considered to address the substantial levels of undiagnosed HIV in high HIV prevalence settings. Community-based HIV testing strategies have shown high acceptability and uptake among adults and likely identify individuals at an earlier stage of the disease sprectrum.[12, 13] However, the yield is lower, which may make community-based approaches less affordable in resource-limited settings. Therefore, strategies that target those at risk of being HIV-infected for HIV testing and counselling are increasingly being considered, particularly in children in whom HIV prevalence is much lower than that in adults.

We previously developed and evaluated a screening tool to identify older children and adolescents attending primary care facilities at risk of vertically transmitted HIV, who would then be targeted for HIV testing.[14] The 4-item screening tool demonstrated a high sensitivity of 80% and halved numbers need to test to identify one HIV-positive child.[7] The validation in the community involved the use of the tool in an asymptomatic population in which the prevalence of HIV was <3% and the disease spectrum of HIV was earlier and included less severe cases. This resulted in the sensitivity to be lower (56% versus 80%) and specificity higher (75% vs 66%) than when the same tool was validated in a hospital setting where children suspected of having advanced HIV were more likely to be found.[7] The main difference was the lower prevalence of the health-related screening items, that is, skin problems and poor health, which are likely to be more prevalent among children attending health facilities with advanced disease. In both settings, orphanhood and previous hospitalisation remained significantly associated with HIV.

Use of the screening tool needs to be weighed against the resources required for universal HIV screening when HIV prevalence is low and the potential benefits. In a community setting with an HIV prevalence of 1.3% to 2.4%, the 4-item tool reduced the number that would need to be tested to identify one HIV-positive child from 76 using universal HIV testing to 34. This is comparable to a reduction from 22 to 10 if the 4-item screening tool was implemented at a healthcare facility level.[7] The prevalence of HIV in this sub-population was <3%, and the high NPV provides assurance that in children who score 0, the probability that they are not living with HIV is 99.2%. However, it is also important to note that the PPV and NPV are dependent upon the prevalence of HIV in the population screened. Although the tool ability to discriminate those at risk or not at risk of HIV was poor (AUC = 65%), the consequences of a false negative or false positive are reasonable. In our population, if the screening tool were applied, 32% of children misclassified as being at risk would have benefited from knowing their HIV status. as It is important to point out that even in the absence of all screening items, HIV testing should be offered to all children if resources are available. However, the tool misclassified 44% of children who were positive as not being at risk, which means this can give a false sense of assurance to nearly half the children living with undiagnosed HIV in need of an HIV test. Therefore, the results of the screening tool should not be considered as definitive and there is need to develop a tool adapted to a community setting.

The findings from this validation study only show that when resources are limited and universal testing is not feasible, this simple, inexpensive and not harmful tool can be adopted for targeted testing to reduce the number needed to test for HIV.

The strengths of the study are a large sample size and high participation rates. The random selection of participants increases the reliability of the study. The results can be generalised to other African settings with similar HIV prevalence. However, there are several limitations to this study. It is possible that some participants may have underreported their HIV status and hence might not have been undiagnosed. OMT tests results were used as a gold standard, but it is an imperfect gold standard as it can give a false negative result in people taking ART, potentially leading to misclassification, and affecting the tool’s sensitivity.[15]

Inclusion of previously diagnosed children would have resulted in spectrum bias and given inflated and potentially misleading estimates of the performance of tool as shown by the increase of the sensitivity of the screening tool from 56% to 71% when previously diagnosed children were included. In addition, the association of a particular screening item with HIV may change over time thereby affecting the performance of the tool, for example, orphanhood levels may drop as ART is scaled up. Further, extrapolation bias might have occurred because the screening tool was developed for 6-16year age group, targeting vertically infected children but was validated in the 8-17year group which might have included horizontally infected children. The screening items used might not have been valid in this population and new screening items might need to be explored, like schooling and sexual health [15]. This might explain the fact that those with a score of 1 were older, 13-17years (32% vs 19%, p<0.001) and there was no significant difference by gender.

## Conclusion

HIV testing and counselling is the critical entry point to accessing HIV treatment. The 2015 WHO guidelines recommend treatment of all individuals living with HIV regardless of age and disease stage.[16] Practical and cost-effective strategies are therefore urgently needed if we are to meet the UNAIDS target to test 90% of all individuals living with HIV by 2020.[17] While universal testing is the gold standard, the use of a screening tool may be a more efficient and potentially a cost-effective strategy particularly among adolescents, an age-group that has a high burden of undiagnosed HIV but a relatively low HIV prevalence and requires checking on only 4- simple items which can be asked by lower level healthcare workers.[6, 8, 18] However, a screening tool will need to be tailored to the community and local context and importantly any testing strategy needs to combine with strategies to facilitate linkage with HIV care for those who test HIV-positive.

## Supporting information

S1 Dataset (XLSX)
